# Pathogenicity of Avian Polyomaviruses and Prospect of Vaccine Development

**DOI:** 10.3390/v14092079

**Published:** 2022-09-19

**Authors:** Chen-Wei Wang, Yung-Liang Chen, Simon J. T. Mao, Tzu-Chieh Lin, Ching-Wen Wu, Duangsuda Thongchan, Chi-Young Wang, Hung-Yi Wu

**Affiliations:** 1 Department of Veterinary Medicine, College of Veterinary Medicine, National Pingtung University of Science and Technology, Pingtung 912, Taiwan; 2 International Degree Program in Animal Vaccine Technology, National Pingtung University of Science and Technology, Pingtung 912, Taiwan; 3 Department of Medical Laboratory Science and Biotechnology, Yuan Pei University of Medical Technology, Yuanpei Street, Hsinchu 300, Taiwan; 4 Department of Biological Science and Technology, National Chiao Tung University, Hsinchu 300, Taiwan; 5 Department of Tropical Agriculture and International Cooperation, National Pingtung University of Science and Technology, Pingtung 912, Taiwan; 6 Faculty of Agriculture and Technology, Rajamangala University of Technology Isan, Surin Campus, Nakhon Ratchasima 30000, Thailand; 7 Department of Veterinary Medicine, College of Veterinary Medicine, National Chung Hsing University, Taichung 402, Taiwan; 8 The iEGG and Animal Biotechnology Center, National Chung Hsing University, Taichung 402, Taiwan

**Keywords:** avian polyomavirus, *Gammapolyomavirus*, genomic structure, pathogenicity, vaccine

## Abstract

Polyomaviruses are nonenveloped icosahedral viruses with a double-stranded circular DNA containing approximately 5000 bp and 5–6 open reading frames. In contrast to mammalian polyomaviruses (MPVs), avian polyomaviruses (APVs) exhibit high lethality and multipathogenicity, causing severe infections in birds without oncogenicity. APVs are classified into 10 major species: Adélie penguin polyomavirus, budgerigar fledgling disease virus, butcherbird polyomavirus, canary polyomavirus, cormorant polyomavirus, crow polyomavirus, *Erythrura gouldiae* polyomavirus, finch polyomavirus, goose hemorrhagic polyomavirus, and Hungarian finch polyomavirus under the genus *Gammapolyomavirus*. This paper briefly reviews the genomic structure and pathogenicity of the 10 species of APV and some of their differences in terms of virulence from MPVs. Each gene’s genomic size, number of amino acid residues encoding each gene, and key biologic functions are discussed. The rationale for APV classification from the *Polyomavirdae* family and phylogenetic analyses among the 10 APVs are also discussed. The clinical symptoms in birds caused by APV infection are summarized. Finally, the strategies for developing an effective vaccine containing essential epitopes for preventing virus infection in birds are discussed. We hope that more effective and safe vaccines with diverse protection will be developed in the future to solve or alleviate the problems of viral infection.

## 1. Introduction

**Figure 1:**
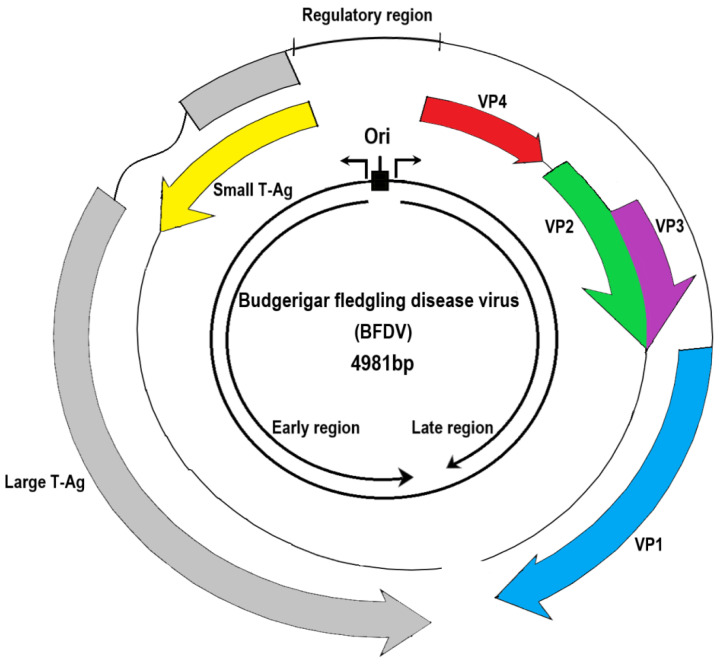
A genomic structure of avian polyomavirus using budgerigar fledgling disease virus as a typical example. In general, all polyomaviruses containing 5–6 open reading frames (ORFs) are composed of an early coding region, which encodes the large T-Ag (colored in gray) and small T-Ag (yellow), and a late region, which encodes structural proteins VP1 (blue), VP2 (green), and VP3 (violet). The VP4 (red) in the genome of the budgerigar fledgling disease virus is not always present in APVs. Some APVs (e.g., butcherbird polyomavirus, cormorant polyomavirus, crow polyomavirus, *Erythrura gouldiae* polyomavirus, finch polyomavirus, goose hemorrhagic polyomavirus, and Hungarian finch polyomavirus) have a putative VP4 or ORF-X protein. The key biological role of each gene is given in Table 1.

Avian polyomaviruses (APVs) have a nonenveloped, icosahedral capsid with a diameter of 40–50 nm and encode a double-stranded circular DNA genome of approximately 5000 bp in length. Because budgerigar fledgling disease virus (BFDV) persistently infects many bird species globally, we used BFDV as a typical example (Figure 1) to describe the common genomic structure of avian polyomaviruses. The genome has early and late coding regions, with a noncoding regulatory control element located between the two regions for DNA replication and gene regulation (Figure 1, top). The early coding region encodes two proteins, large tumor antigen (large T-Ag) and small tumor antigen (small T-Ag), while the late coding region encodes structural proteins VP1, VP2, and VP3. Some polyomaviruses have a putative VP4 or a putative open reading frame (ORF) X that is located between the start and stop codons. In BFDV, the late coding region expresses VP4, and the VP4 ORF contains genes for VP4 and VP4 delta [1,2]. APVs have 5–6 ORFs depending on the species. The number of amino acid residues of putative ORF-X products may have functional similarities with the VP4 of BFDV. Using BFDV as a typical example, the physiologic properties of each gene are listed in Table 1.

In 2015, the International Committee on Taxonomy of Viruses conducted a comprehensive revision of polyomavirus taxonomy in order to clearly define virus species and created a nomenclature using the combination of host name and viral genomic sequence. Even though the virus name is used to highlight the host specificity, there are few APVs including BFDV, finch polyomavirus (FPyV), and goose hemorrhagic polyomavirus (GHPyV) that can infect many other birds [12,13,14,15,16].

**Figure 2:**
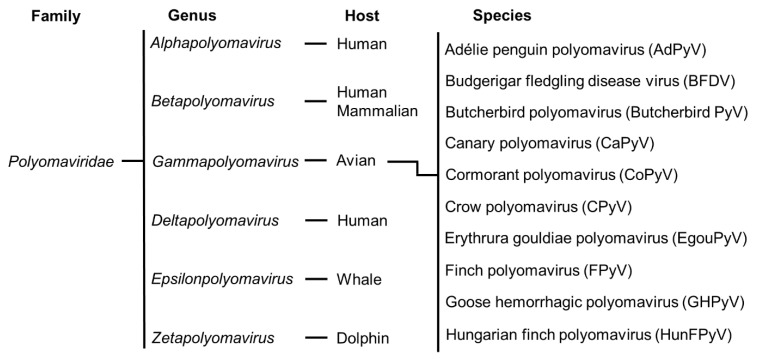
Classification of APVs under the genus *Gammapolyomavirus*. The *Polyomaviridae* family is generally divided into six genera. There are 10 APVs that belong to the genus *Gammapolyomavirus*.

In 2017, based on gene differences among various viral proteins, the International Committee proposed using the relatively conserved large T-Ag amino acid sequence as a base for the classification of polyomavirus genus and species [12,17]. The family *Polyomaviridae* is classified into six genera. All the APVs mentioned in this review under the genus *Gammapolyomavirus* and their classification with the MPVs are illustrated in Figure 2.

The *Polyomaviridae* family is generally divided into six genera: *Alphapolyomavirus*, *Betapolyomavirus*, *Gammapolyomavirus*, *Deltapolyomavirus*, *Epsilonpolyomavirus*, and *Zetapolyomavirus* [12,18]. Different polyomavirus genera usually infect different types of animals with some host specificity: alpha, beta, and delta polyomaviruses mostly infect humans and other mammals, while gamma, epsilon, and zeta polyomaviruses infect birds, whales, and dolphins, respectively. Further, the study by Burck et al. (2016) revealed that some polyomavirus-like sequences detected by the shot-gun approach were found in several fish species, but they are not classified as polyomaviruses [19].

Under the genus *Gammapolyomaviruses*, there are 10 APVs officially identified as Adélie penguin polyomavirus (AdPyV), BFDV, butcherbird polyomavirus (Butcherbird PyV), canary polyomavirus (CaPyV), cormorant polyomavirus (CoPyV), crow polyomavirus (CPyV), *Erythrura gouldiae* polyomavirus (EgouPyV), FPyV, GHPyV, and Hungarian finch polyomavirus (HunFPyV) [2,17,20,21,22]. The nucleotide size and the number of amino acid residues corresponding to each gene and its potential biological role using BFDV as a typical example are listed in Table 1.

## 2. Major Pathogenic Difference between Avian and Mammalian Polyomaviruses

MPVs often cause subclinical or asymptomatic infection in their natural hosts; some symptoms appear only in severely immunocompromised hosts. Interestingly, these viruses can live in their hosts for life [23]. Compared to MPVs, APVs have unique biological characteristics, including a broad host range, substantial pathogenicity, and a preference for tissue tropism [24]. In addition, VP4 is relatively important in APV infection and is thought to be associated with viral genome packaging and cell apoptosis in cell cultures (Table 1). A previous study showed that deleting or inactivating VP4 attenuates BFDV virulence [1,10]. Another reason MPVs, such as JC polyomavirus, are less or not lethal in terms of pathogenicity is that they may induce the expression of interferon (IFN)-α and IFN-β (two components responsible for host innate immunity) in various human primary cells, thereby inhibiting their own replication [25,26]. On the contrary, in an ex vivo study, it showed that APV replicated efficiently in chicken embryo fibroblast cells. It also showed that BFDV proteins (VP4 and VP4 delta) could directly bind to the IFN-β promoter and downregulate the expression of IFN-β, allowing the virus to replicate in host cells. It provides the molecular mechanism by which BFDV can escape the host’s innate immunity [27].

## 3. Clinical Signs and Disease Progression of Avian Polyomavirus Infection

An overview of the 10 APV species with respect to their hosts and the clinical symptoms that they cause is summarized in Table 2.

In 1981, BFDV was reported in budgerigars and identified as the pathogen responsible for causing BFD. The genomic structure of BFDV is depicted in Table 1 [1,2]. The signs of BFDV infection symptoms include hepatitis, ascites, pericardial effusion, and abdominal distension [30,31]. Adult and chronically infected parrots often exhibit abnormal feather growth [39]. The virus is shed in feces for up to 6 months after infection [40]. In addition, budgerigars are suspected to be a bird species that can be persistently infected with this virus and existed for life. However, another study showed that the progeny of serum-positive adult budgerigars housed in a completely clean and BFDV-free environment for 7 months before breeding are clinically normal without the presence of antibodies [40]. BFDV has no strict host specificity and can infect many birds, including chicken, vulture, falcon, canary, ostrich, pigeon, duck, goose, finch, gull, common raven, pheasant, Eurasian jay, and starling [30,41,42]. According to the literature, BFDV infection has been observed globally in parrots in Canada, Australia, Germany, the US, Switzerland, Slovakia, Italy, Japan, Taiwan, China, Thailand, Costa Rica, Turkey, Pakistan, Peru, and Chile [43,44,45,46,47,48,49,50,51,52]. Alignment with viral genomic sequences from Asia, North America, Europe, and Australia revealed that BFDV is widespread globally. The lack of host specificity is concerning as it may both transmit and contribute to the virus being widely spread. In addition, the possible routes of BFDV transmission, including bird migration, free trade in poultry, and bird transportation, have been highlighted and discussed by Kou et al. [49].

GHPyV is the pathogen responsible for hemorrhagic nephritis and enteritis in geese, which has a prevalence rate of 4–67% in 3–10-week-old geese [15,34]. A study conducted in 2004 found that neurological symptoms appeared in 1-day-old goslings after GHPyV inoculation, with a 100% mortality rate [35]. The full-length genomic DNA of this virus is approximately 5252–5256 bp [53]. The disease in geese was first reported in Hungary in 1969, and the main symptoms included subcutaneous edema, ascites, renal pallor, and swelling, with occasional gastrointestinal hemorrhage [15]. Histological examinations commonly reveal renal and intestinal vascular necrosis, bleeding, and edema [15]. In acute cases, hemorrhagic necrosis is observed in the small intestine as well as in the endothelial and vascular walls of blood vessels. It is often accompanied by vasculitis with bleeding and inflammatory cell infiltration as well as occasionally by mucosal bleeding in the proventriculus and gizzard. Necrotizing vasculitis is observed in subacute or chronic cases. In term of renal lesions, distal renal tubule necrosis is more severe than proximal tubule necrosis. Uric acid deposition, calcification, and fibrosis occur as the disease progresses are responsible for the gout formation. These symptoms are more severe in subacute and chronic cases than in acute cases [35,36,37]. In addition, in situ hybridization results revealed that GHPyV is primarily found in the nucleus and cytoplasm of endothelial cells in systemic arteries, veins, and capillaries, but it is absent in cells from other tissues. This also explains why GHPyV infection results in anemia, subcutaneous edema, ascites, or intestinal bleeding. Furthermore, it damages vascular endothelial cells, resulting in vascular dysfunction, thereby increasing vascular permeability and causing edema, hematoma, vessel rupture, and bleeding [37,54]. Although it was previously thought that GHPyV caused lymphoid depletion in the spleen and bursa of Fabricius, the in situ hybridization conducted in 2021 suggested that GHPyV is not present in the lymphocytes of these organs [35,36].

Initially, GHPyV was predominantly found in Europe, with the clinical cases of hemorrhagic nephritis enteritis associated with geese [35]. In 2008, it was found that GHPyV could infect ducks [19]. Currently, it is believed that geese that have survived GHPyV infection can continuously transmit this virus, while ducks are asymptomatic carriers [30,34,36,38,55]. Subsequently, in 2018, GHPyV was identified in ducks across China via polymerase chain reaction; however, it was not identified in geese [56]. In 2021, the first case of this disease was reported in Taiwan from geese outside of Europe [37]. Furthermore, GHPyV has been suspected of being able to infect geese indefinitely [55].

The genomic sequence of FPyV from Eurasian bullfinches isolated in 2006 revealed that it is remarkably different from BFDV and was thus named finch polyomavirus [2]. The FPyV genome is 5278-bp long [2]. Subsequently, identical or similar FPyV sequences were reported in European goldfinches [57], grey-headed bullfinches [58], and Gouldian finches [13,22]. Earlier research had identified polyomavirus infections in various finches; however, there was no way of determining whether these infections were caused by FPyV or BFDV. Examples include canaries, European goldfinches, European greenfinches, and other Fringillidae birds [59,60,61], as well as Gouldian finches, seed crackers, western bluebills, tricolored munias, cordon-bleu finches, and painted finches [14,47,62]. Infected finches developed typical lesions, such as liver necrosis, membranous nephropathy, and liver and renal cell nuclei hypertrophy, in which a large number of transparent-to-basophilic intranuclear inclusion bodies were found but not in periarteriolar lymphoid sheaths [63].

In 2006, a study on the clinical outcomes of CPyV was conducted [2]. Spleen samples of dead wild western jackdaws collected in northeast Spain in early 2005 were found to carry this virus. The genome of this virus is 5079-bp long [2]. Clinically, it was speculated that the death of jackdaws from CPyV was mainly associated with Salmonella co-infection [2].

CaPyV was discovered in 2010 as a result of a decrease in egg laying and hatching rates of canaries. The offspring of the animal were found dead at 40 days of age, with a 50% mortality rate. The genomic DNA of this virus is 5421-bp, and the most critical difference between CaPyV and other APVs is that CaPyV does not express VP4. VP4 promotes apoptosis in host cells and accelerates the infection rate [1]. It appears to be controversial because CaPyV lacking VP4 can still cause gross lesions, including subcutaneous bleeding and hepatosplenomegaly, in host animals. In the liver and spleen, there was extensive centrilobular degeneration with hemorrhage and intranuclear inclusion bodies. Some inclusion bodies were found in the renal glomerular epithelium [32].

Butcherbird PyV is 5084-bp long. The clinical symptoms of Butcherbird PyV differ from those of typical APVs. The source of the virus was accidentally discovered in wild adult grey butcherbirds with no apparent signs other than periocular nodule growth. It is unclear whether these symptoms in the periocular nodule were related to Butcherbird PyV; the animal recovered after the nodule was resected [29].

AdPyV was discovered in the Cape Crozier colony on Ross Island in Antarctica in 2014, primarily causing feather loss in Adélie penguins [28]. Its genomic DNA is 4988-bp in length [28]. Varsani et al. found that AdPyV has a direct relationship with Adélie penguins in that south polar skuas prey on Adélie penguin eggs and fledglings; therefore, south polar skuas may carry the polyomavirus and spread it in the environment of the South Pole [64].

EgouPyV was discovered in Gouldian finches (*Erythrura gouldiae*) and reported in 2015. The genome of the virus consists of 5172-bp, and its nucleotide sequence has the highest homology (71%) with CPyV but only 41% with Butcherbird PyV. The associated clinical signs have not yet been thoroughly reported [20].

HunFPyV was first discovered in white-headed munia (*Lonchura maja*). The virus genome is 5284-bp long and shares 91% homology with FPyV. In EgouPyV (the other FPyV), the values were less than 65%. Clinically, liver failure, nephritis, and myocarditis are associated with the affected birds [22].

**Figure 3:**
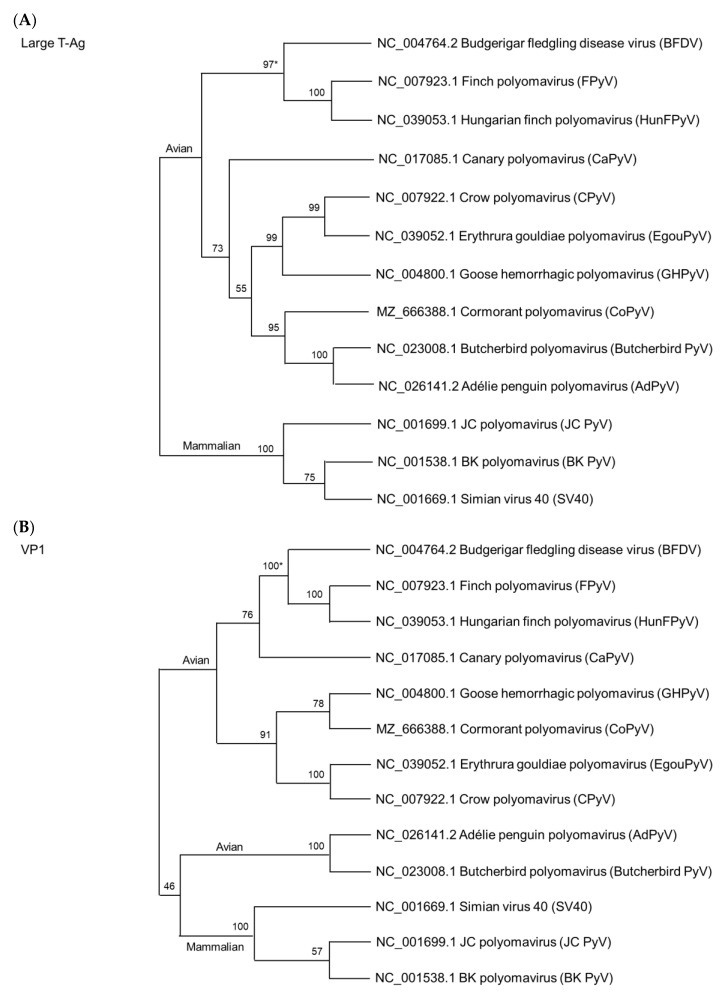
Phylogenetic analyses using amino acid sequences of the large T-Ag (**A**) and VP1 (**B**) from the *Gammapolyomaviruses.* The phylogeny, which uses the large T-Ag, can distinguish between APVs and MPVs. Interestingly, using VP1 for the analysis, Butcherbird PyV and AdPyV show the closest relationship with MPVs on a common branch. Pathogenically, their clinical symptoms are mild and there is no mortality (Table 2) [28,29]. Virus names and GenBank accession numbers are shown on the phylogenetic tree. * The bootstrap value on the branch represents the percentage while using 1000 bootstrap replicates.

Most recently, in 2022, Feher et al. reported a novel polyomavirus called cormorant polyomavirus (CoPyV) in great cormorants (*Phalacrocorax carbo*) [21]. The DNA is 5133-bp in length and has a genome similar to that of GHPyV (Figure 3). There were no detailed clinical signs reported [21].

## 4. Some Molecular Characteristics Involved in the Pathogenicity of Avian Polyomaviruses

As shown in Figure 1, the polyomavirus genome is divided into two regions: the early region, which encodes the large T-Ag and small T-Ag, and the late region, which encodes the structural proteins VP1, VP2, VP3, and VP4; a noncoding control element is present between these two regions [1,2]. The noncoding control region contains a bidirectional replication domain with an element for binding virus and host cell transcriptional factors to regulate the expression of early and late genes of the virus [65]. In all BFDV genomic sequences, two sets of the unique palindromic tandem repeats (TCC(A/T)_6_GGG/A) are present in the noncoding control region, while NCC(A/T)_6_GGN is present in GHPyV, CPyV, FPyV, and CaPyV [2,66]. Further studies on the palindromic sequence of BFDV reveal that this tandem repeat may bind to the large T-Ag. The nuclear localization sequence in large T-Ags could specifically bind to the replication region in the noncoding control region. In BFDV and FPyV, nuclear localization sequences contain both lysine and arginine residues, whereas in GHPyV, CPyV, and CaPyV, they contain only lysine. Insertion, deletion, and point mutations in the noncoding control region are thought to be one of the main and critical mechanisms by which polyomaviruses change their adaptive behavior, such as host jumping [67]. In short, the main function of large T-Ag in APVs is to interact with tandem repeat sequences in the noncoding control region to regulate DNA replication and RNA transcription for the virus [3,4,68].

BFDV has been proven to infect many bird species. These animals can also be simultaneously co-infected by other APVs [30,41,42]. Related studies found that one MPV species could recombine with other MPV species in the host animal. For example, a 2017 study demonstrated that African bat mammalian polyomavirus might recombine in the bat. This recombination is usually associated with hotspots that are expressed for viral capsid proteins [67]. As mentioned above, the polyomavirus VP1 is involved in host cell recognition [69], and thus recombination could alter the specificity for host transmission. In 2017, Carr et al. showed that recombination events in polyomavirus evolution require a long period of time [67]. As recombination hotspots include the domains of capsid proteins (e.g., VP1), it may increase in changing the host preference and in the probability of species jumps. It may also make the APV infection more complex and concerning.

In MPV, the large T-Ag has functionally conserved domains, such as the DnaJ-, LXCXE, nuclear localization sequence, helicase, and p53-binding domain, which are found in most polyomaviruses [70]. One study reported that these domains can bind to tumor suppressors in the host (p53 and Rb), thereby promoting cell cycle changes to enhance tumorigenesis [71]. In contrast, these mechanisms are rarely observed in APV. APVs induce apoptosis in host cells during infection, and this mechanism is thought to effectively spread progeny viruses, resulting in different virulence [11]. Analysis of the complete sequence of DnaJ and helicase domains of the large T-Ag gene revealed that these domains play important roles in the biochemical function of the large T-Ag and that these domains are relatively stable among different polyomaviruses compared to other domains [72].

The small T-Ag shares an amino-terminal domain with the large T-Ag because both proteins originate from the same ATG codon [5]. Because the gene sequences of large T-Ag and small T-Ag overlap to some extent (Figure 1), the two proteins share some functional similarities. The small T-Ag in MPV also exhibits cell transformation potential in tissue culture and is tumorigenic in experimental animals [6,7]. In 2022, a study on the sequence analyses of all available APV isolates in Taiwan found that the large T-Ag was the most conserved protein, whereas the small T-Ag was the least conserved [66,73].

VP1 is a major capsid protein whose primary function is to bind to sialylated glycan receptors of host cells. In virus-like particles of Simian virus 40, VP1 forms a pentamer and is stabilized by calcium ions and disulfide bonds [74,75]. In the cavity of the pentamer, a single copy of VP2 or VP3 binds to VP1 in a hairpin-like manner [76,77,78,79]. VP1 recognizes host cells primarily through its specific determinant, of which glutamic acid residue at 92 is the most important for host cell recognition. It plays an essential role in viral tropism, transmission, and cell targeting [69]. VP1 requires a 12-amino-acid fragment to bind to a calcium ion to promote capsid formation. This sequence in BFDV is 237-DENGVGPLCKGD-248 and differs from that of MPV by only one amino acid residue [80,81]. The study showed that VP4 of BFDV and ORF-X of GHPyV have the highest variation in APVs [66,82]. The VP4 and VP4 delta proteins suppress host immune responses by inducing apoptosis. Variations in these proteins may thus affect the pathogenicity of BFDV [30,66,73]. The VP4 protein is enriched with basic amino acids located in the central region between residues 70 and 77 to mediate DNA binding but not for that of VP4 delta. In addition, VP4 may stabilize itself via dimerization before binding with DNA [22].

## 5. Phylogenetic Relationship among Avian Polyomaviruses

In 2021, Kaszab et al. conducted a comprehensive study on APV evolution. They calculated the evolutionary rates of APV in substitutions/nucleotide site/year (s/s/y) [82]. The mean evolutionary rate of BFDV is 1.39 × 10^−4^ s/s/y (7.18 × 10^−5^ – 2.10 × 10^−4^ s/s/y), which is 10 times faster than that of GHPyV (mean 3.03 × 10^−5^ s/s/y; 1.09 × 10^−5^ – 5.33 × 10^−5^ s/s/y) but similar to those of FPyV (mean 2.63 × 10^−4^ s/s/y; 1.60 × 10^–8^ – 6.26 × 10^–4^ s/s/y) and CaPyV (mean 1.41 × 10^−4^ s/s/y; 5.75 × 10^−10^–7.17 × 10^−4^ s/s/y) [82].

Using large T-Ag and VP1 as a base, we performed the phylogenetic analyses using Molecular Evolutionary Genetics Analysis version 11 (MEGA, https://www.megasoftware.net in assessed date 1 July 2022) in which the Clustal W method was used for amino acid sequence alignment, while the maximum likelihood method was employed with the LG model for large T-Ag and VP1 [83]. The sequence of each GenBank accession number is indicated in Figure 3.

As shown in Figure 3A, we used the large T-Ag amino acid sequences from 10 APVs as a model to analyze their phylogenetic relationship because the sequences are conserved and have been utilized as the key protein for APV classification [12]. As expected, there is a significant distance in their relationship between the avian and mammalian polyomaviruses, which is consistent with previous views on their structural differences [23,24]. As shown in Figure 3B, we used the VP1 protein for the phylogenetic analyses. It showed a unique result. The Butcherbird PyV and AdPyV have a closer relationship with three MPVs than with other APVs. Currently, it is unclear whether the sequence similarity identified in the phylogenetic relationship can be used as a reference to determine some partial similarity of pathogenicity among the virus species. Additional studies with detailed pathogenicity among the species are needed because of the limited studies conducted thus far.

## 6. Historical Development of Vaccines for Avian Polyomaviruses

In terms of vaccine development, some studies on BFDV have been conducted [84,85,86,87]. In 1996, an inactivated vaccine against BFDV was developed using a BFDV isolate [84]. The vaccine was safe, capable of neutralizing viruses, and protected the host animals from infection [84]. In 1998, a study was conducted to further examine the safety, neutralizing capability, and efficacy of the viral vaccine in parrots (non-budgerigar) of different ages and species. The results revealed that the vaccine effectively prevents BFDV from infection [88].

The vaccine containing inactivated BFDV was effective, but there were no subsequent follow-ups to determine the long-term efficacy in terms of host immunity [88]. Because Kaszab et al. showed that the mean evolutionary rate of BFDV is 1.39 × 10^−4^ s/s/y, with the VP1–VP3 genes evolving at the fastest rates, the protection efficacy of previously developed vaccines would be potentially attenuated with the emergence of new infected strains [82]. In addition, this vaccine was mainly prepared using primary chicken embryo fibroblast in cell culture, which is time-consuming, costly, and requires advanced techniques for production.

Compared to poultry, parrots are more prone to stress and exhibit highly aggressive behavior, making subcutaneous injection difficult. Injections may be feasible for personal pets, but it is extremely difficult to inject a large number of parrots on a farm. Under this situation, it would be better to use alternative inoculation methods (such as intranasal administration, nebulization, and oral feeding via drinking water) with inactivated viral particles. Therefore, attenuation of virulence or deletion of major virulent determinants by creating a live nonlethal virus is needed. For example, fiber-2 was deleted from highly pathogenic fowl adenovirus serotype 4 in 2022 to protect against this fatal viral infection [89]. Another strategy has been tested in which a nonlethal virus was inserted with a lethal viral structure protein. A vaccine against highly lethal avian bornavirus was developed using recombinant virus to protect parrots from Newcastle disease virus [90,91,92].

An early GHPyV vaccine-related study revealed that this virus cannot proliferate in cell culture. The viral vaccine was then prepared using the recombinant technique to express viral protein in yeast or Sf9 insect cells [85,93]. Effective antibodies prepared against specific epitopes of GHPyV were successfully raised in geese, demonstrating that this vaccine can induce antibodies in birds [85]. In a recent article, GHPyV production has been successfully propagated in cultured primary goose embryo fibroblasts, showing the availability of information regarding the gene expression profile [87]. This should allow for the future preparation for the GHPyV vaccine when needed.

Another study was conducted using GHPyV vaccines prepared from a recombinant virus expressing the VP1 gene under the control of the polyhedron promoter in insect cells. After two vaccinations at days 1 and 18, the survival rate was 100% against a challenge at week 5. [85]. In 2010, Gelfi et al. found that using Al(OH)_3_ as an adjuvant for vaccine preparation resulted in poor immunogenicity, whereas the vaccine preparation using acrylate polymer carbopol mixed with β-propiolactone-inactivated GHPyV was safe and immunogenic [84]. Thus, the adjuvant also plays a crucial role in the development of an effective vaccine. Interestingly, this vaccine also induces the transfer of maternally derived neutralizing antibodies to goslings, which can still be detected 15 days after hatching, indicating that the vaccine effectively protects goslings from GHPyV infection [86]. Although low-cost Sf9 insect cells were used to express the VP1 protein [85], the overall cost of producing vaccines is still high. Therefore, it is difficult to commercialize these two vaccines for farm animals. The lack of commercially available vaccines may explain why many GHPyV cases continue to occur in Europe [33,34,53,87].

The virus-like particles are commonly used in the development of single-virus vaccines [85,93,94,95,96,97]. Regardless of vaccine cost, the capsid protein VP1 is successfully expressed in eukaryotic systems in sufficient quantities for vaccine preparation [98]. The development of virus-like particles of APV began in 2002, when Sasnauskas et al. used insect and yeast cells to express recombinant proteins of BFDV, thereby encouraging the preparation of the VP1 subunit as a vaccine for GHPyV in 2006 [93,98]. It also revealed that, with the exception of FPyV, the receptor-binding mechanisms of BFDV, GHPyV, and CPyV are similar [94]. We anticipate that effective polyvalent vaccines will be developed once the molecular structure of different APV species is better understood.

It has been further hypothesized that some of the receptors, such as the sialylated glycan receptor, have specifically evolved within polyomaviruses. Because the binding mechanisms for VP1 are similar across APV species, there is some immunochemical cross-reactivity [2,15,99]. This could be used to look for the VP1 protein of APVs as well as other receptor-binding domains for the epitope-based vaccine [100,101]. Polypeptides containing multiple APV epitopes may thus provide comprehensive protection compared to subunit fragments alone. It adds a new dimension to vaccine preparation, making it more flexible and effective. For example, Wang et al. (2017) used a multi-variant epitope ensemble vaccine to raise antibodies against an avian leucosis virus subgroup J [100].

## 7. Some Prospects with Respect to the Development of Vaccines Utilizing the Epitope of Receptor-Binding Domain

It makes sense to develop a vaccine that generates antibodies capable of directly inactivating or blocking the viral receptor-binding domain involved in the recognition between the virus and host cells. This functionally based epitope would be an excellent target for vaccine development. However, using monoclonal antibodies prepared against human low-density lipoprotein (LDL), Mao et al. first demonstrated that the binding of one specific monoclonal antibody to LDL can drastically enhance the binding of another specific monoclonal antibody via cooperative binding. This enhancement is not additive but synergistic, with a 10-fold increase in binding to LDL [102,103]. It has also been discovered that a single monoclonal antibody can even precipitate LDL by altering its conformation, presumably by exposing the hydrophobic region of the apolipoprotein of LDL [104]. Notably, LDL, one of the largest proteins in human plasma, has a diameter of approximately 30 nm, which is comparable to those of avian polyomaviruses (40–50 nm). The current life-threatening pandemic virus, SARS-CoV-2, is approximately 120 nm in size, which is four times larger than LDL. Because of the constant mutations in SARS-CoV-2 [105], it is clear that a new strategy for developing a new generation of vaccines using additional epitopes other than those epitopes involved in the receptor-binding domain is required. Some considerations are as follows:

1. One monoclonal antibody can enhance the binding of another monoclonal antibody via cooperative binding, as proposed by Mao et al. (1983) [102,103]. This should not be limited to two antigenic domains because each epitope is relatively small, containing only approximately seven amino acid residues [106].
2. The selection of a specific region of a viral protein for vaccine preparation should not only be made against a functional domain, but other structural proteins with “constant” regions or regions with minimal mutations should also be considered as a target to stabilize the antigen–antibody interaction. Other region(s) that will cause a drastic conformational change in the virus structure could also be considered as targets, such as precipitating antibodies found against LDL [104] that could directly immobilize viral activity.
3. Finally, because the genomes of the 10 major avian polyomaviruses are relatively small, containing only six genes, it is possible to prepare a “cocktail” multivalent vaccine to produce “APV-capturing antibodies” within a single preparation.

## 8. Conclusions

APVs have a broad host range appearing to be almost distributed worldwide and may be highly pathogenic (Table 2). Although there is currently no evidence that an APV will recombine with another APV species, awareness must be maintained because APVs cause high mortality in birds. Using the VP1 protein sequence for phylogenetic analyses, both Butcherbird PyV and AdPyV of APVs and MPVs have low pathogenicity in terms of developing clinical symptoms and they are closely related on the phylogenetic tree (Table 2). It is unknown whether these two avian species will cause cross-species infections between avians and mammals. Nevertheless, the Newcastle disease virus found in fowls has been confirmed to be transmissible to humans, causing fatal encephalitis in a child [107].

Migration of birds is not the only route through which viral infections can spread globally. The overlap among bird transportation, intensive farming, the existence of wild animals, human routine travel activities, and genome exchange may increase the chances of viral infection and pandemics. Therefore, preventive measures must be implemented to prevent viruses from spreading among susceptible animal hosts [82]. In addition, breeder biosafety assays, pathogen testing, and effective quarantine measures can also be involved in preventing infection by APVs [108].

Vaccines still play an indispensable and reliable role in the prevention of viral infection. In the APV vaccine study, the traditional, subunit, and inactivated vaccines and virus-like particles were developed. However, owing to the recent outbreak of SARS-CoV-2 (CoV-19 infection), the most recently developed mRNA-based vaccine has received much attention and has demonstrated remarkable efficacy in humans to protect against coronavirus infection [105].

Regardless of the techniques already developed for APV vaccine preparation, an mRNA-based vaccine could be another option in APV vaccine preparation because it is convenient and safe to synthesize without causing too much biohazard. The information described in Section 6 is also essential for providing the basis and strategy for future mRNA-based vaccine designs. They can also provide the specific protein motif containing the epitope in the receptor recognition determinants without using tissue culture to prepare recombinant protein [109]. The desired synthetic mRNA may not be restricted to the epitope located within the host recognition site but may also include other epitopes within structural proteins or other functional proteins that can enhance the binding of antibodies via cooperative binding [101,103] or immobilize the virus via precipitation [104]. Although it has not been applied to animals, it provides us with the opportunity to utilize such technology and experience in the future development of bird vaccines containing the key domain with virus recognition [109] and the domains undergoing drastic conformational changes upon binding of specific antibodies from a given host [102,103,104]. If the overall cost is reasonable, it could also produce a cocktail vaccine by combining with several desired mRNAs corresponding to several avian polyomaviruses. Furthermore, mRNA can be designed to trigger specific T cell or B cell recognition in order to optimize the immune response in a short time [110,111]. We hope that more effective and safe vaccines with diverse protections will be developed in the future to alleviate the problem of viral infection.

## Figures and Tables

**Table 1:** Name of the genes with respect to their nucleotide size, amino acid residues, and functions of Avian Polyomaviruses.

	Nucleotide Size (bp)	Amino Acid Residues	Functions [Reference]
Gene: gp5 Protein: Large T-Ag	1995 (1995–2166) *	599 (599–660) *	Interacts with the tandem repeat sequences in the noncoding control region to regulate DNA replication and RNA transcription [3,4]
Gene: gp6 Protein: Small T-Ag	483 (483–537)	145 (145–178)	The large T-Ag from the same ATG codon shares an N-terminal domain [5] and is involved in cell transformation and tumorigenicity [6,7]
Gene: gp4 Protein: VP1	1032 (1032–1083)	343 (343–360)	VP1, a capsid protein, binds to the host cell receptor for infection and forms a pentamer for its stability [8,9], which is further reinforced with a single copy of VP2 and VP3.
Gene: gp2 Protein: VP2	1026 (981–1110)	341 (331–369)	
Gene: gp3 Protein: VP3	708 (654–738)	235 (217–245)	
Gene: gp1 Protein: VP4	675 (485–755) except canary polyomavirus and Adélie penguin polyomavirus	176 (112–205) except canary polyomavirus and Adélie penguin polyomavirus	Suppresses immune responses, induces apoptosis, and increases in pathogenicity [1,10,11] and scaffolding function during virion assembly [1].
Gene: gp1 Protein: VP4d	675	112	VP4 delta (deleted a.a. 69–132 in VP4) contains a leucine zipper-like motif [10]. Induces cell apoptosis and affects releases of viral progeny [1].

* BFDV is used as a typical example. Numbers in parenthesis indicates the ranges in other nine APVs.

**Table 2:** Overview of avian polyomaviruses with their hosts and clinical symptoms developed.

Virus	Host	Clinical Symptoms [Reference]
Adélie penguin polyomavirus (AdPyV)	Adélie penguins (*Pygoscelis adeliae*)	Feather loss [28]
Butcherbird polyomavirus (Butcherbird PyV)	Grey butcherbird (*Cracticus torquatus*)	No apparent signs were observed except for periocular nodule growth. It is unclear whether these symptoms are directly associated with Butcherbird PyV [29]
Budgerigar fledgling disease virus (BFDV)	Parrots, chickens, vultures, falcons, canaries, ostriches, pigeons, ducks, geese, finches, gulls, common ravens, pheasants, Eurasian jays, and starlings	Development of hepatitis, ascites, pericardial effusion, and abdominal distension [30,31]; abnormal feather growth often occurs in adult and chronically infected parrots
Canary polyomavirus (CaPyV)	Canaries (*Serinus canaria*)	Subcutaneous bleeding; patosplenomegaly; extensive centrilobular degeneration with hemorrhage in the liver; splenic depletion; or polyomavirus-like intranuclear inclusion bodies in the liver and spleen, occasionally found in the epithelium of some renal glomeruli [32]
Cormorant polyomavirus (CoPyV)	Great cormorant (*Phalacrocorax carbo*)	Detection in the liver, but detailed pathological findings are not available [21]
Crow polyomavirus (CPyV)	Western jackdaws (*Corvus monedula)*	Enteritis and death with Salmonella co-infection [2]
*Erythrura gouldiae* polyomavirus (EgouPyV)	Gouldian finch (*Erythrura gouldiae*)	Detection in the liver, but detailed pathological findings are not available [20]
Finch polyomavirus (FPyV)	European goldfinches, grey-headed bullfinches, and Gouldian finches	Liver necrosis, membranous nephropathy, liver and renal cell nuclei hypertrophy, large amounts of transparent-to-basophilic intranuclear inclusion bodies, with no inclusion bodies in periarteriolar lymphoid sheaths [13,14]
Goose hemorrhagic polyomavirus (GHPyV)	Geese and ducks	Subcutaneous edema, ascites, renal pallor, and swelling; gastrointestinal hemorrhage in some cases [15,33,34,35]; in acute cases, hemorrhagic necrosis in the small intestine, with endothelial and vascular wall necrosis in the proventriculus, gizzard, and intestines; inflammatory cell infiltration, with mucosal bleeding in gizzard; in subacute and chronic disease cases, gout [35,36], renal and intestinal vascular necrosis, bleeding, and edema [15,33,35] are seen; distal renal tubule necrosis is more severe than proximal tubule necrosis, resulting in urate deposition, calcification, and fibrosis [36,37]; ducks are considered asymptomatic carriers [16,38]
Hungarian finch polyomavirus (HunFPyV)	White-headed munia (*Lonchura maja*)	Liver failure, nephritis, and myocarditis [22]

## Data Availability

All the data are summarized from the references listed.
